# Quality of life in Parkinson`s Disease

**Published:** 2012-12-25

**Authors:** JA Opara, W Brola, M Leonardi, B Błaszczyk

**Affiliations:** * Academy of Physical Education in Katowice, Poland; ** District Hospital in Konskie, Poland; *** Fondazione IRCCS, Istituto Neurologico Carlo Besta, Milano, Italy; **** Higher School of Economics and Law, Kielce, Poland

**Keywords:** amelioration, disability, fatigue, Parkinson`s Disease, Quality of Life

## Abstract

In this review report, current possibilities of evaluation of quality of life in Parkinson’s disease have been critically presented. Health Related Quality of Life (-HRQoL) comprises a wide spectrum of consequences of the disease. Measurement of quality of life has become increasingly relevant as an outcome parameter, especially in long-term trials. Most of the available QoL instruments depend on patient self-reports. The data can be collected by written questionnaires. There are universal questionnaires of QoL – for many diseases and the specific ones – specially created for one disease. Among universal questionnaires, the Sickness Impact Profile (SIP) and the Short-Form Health Status Survey (SF-36) are the most popular in Parkinson’s disease. As for specific questionnaires: the Parkinson`s Disease Questionnaire (PDQ-39) and the Parkinson`s Disease Quality of Life Questionnaire (PDQL) have been described.

## Current possibilities of evaluation of Quality of Life in Parkinson`s Disease

Measurement instruments in health research and clinical practice are used to assess the health status of individuals, usually with the objective to make a diagnosis or predict future developments (predictive measures), to evaluate changes in health status over time (evaluative measures) or to discriminate between patients (discriminative measures), for instance with respect to length, weight, or the severity of dyskinesias. Measurement involves the assignment of values to variables and can be performed with various types of instruments, such as questionnaires, structured interviews, tests, or rating scales [**1**].

An overall aim of treatment in Parkinson`s Disease (PD) is to lower the negative impact of the disease on functioning and quality of life of patients. Therefore, a measurement of functioning and quality of life should be included in the evaluation of the effectiveness of treatment. 

The most commonly used quality of life questionnaires, either generic or specific, were presented in this paper. Information about clinical and functional status is useful in the interpretation of the quality of life assessment results. Because of that, instruments for the assessment of depression, cognitive functions, functional ability and fatigue in PD were also described.

## Quality of Life in Parkinson`s Disease

Quality of Life (QoL) is a multi-dimensional construct, which consists of at least three broad domains: physical, mental and social. In the field of medicine, researchers and physicians have often used health-related quality of life concept, which specifically focuses on the impact of an illness and/or treatment on patients’ perception of their status of health and on subjective well-being or satisfaction with life [**2**]. We have described the Quality of Life of post-stroke patients and their caregivers in our first report [**3**], Quality of Life in Multiple Sclerosis [**4**] and QoL in Alzheimer Disease [**5**].

Parkinson`s Disease (PD) can cause a variety of symptoms. Early in the course of the disease, the most obvious symptoms are movement-related, including shaking, rigidity, slowness of movement and difficulty with walking and gait. Later, cognitive and behavioural problems may arise, with dementia commonly occurring in the advanced stages of the disease. Other symptoms including sleep and emotional problems, depression, difficulties in coordination and speech, severe fatigue, problems with balance and pain will have an impact on the patient`s QoL [**6**]. We must also take into account complications caused by treatment with levodopa, like dyskinesias, dystonias, and fluctuations. 

QoL measures are suitable as well for an outcome measure of a new treatment such as rehabilitation [**8-13**]. Subjective factors in QoL in PD patients include perception of symptoms, level of fitness, self-image, satisfaction with family life, work, the economic situation, the interaction with other people, social support, and life in general. To the objective factors, we should include the clinical picture of disease, social status, social and living conditions and the number and intensity of social contacts. The scales used to assess the QoL in PD include either subjective or objective indicators, or both [**8,14**]. The questionnaire may be completed by the patient in person or by telephone interview, family members, or close persons, by the professional carers and health professionals. The most desirable and reliable is the assessment by the patient himself, especially when the subjects of measurement are subjective aspects of QoL. QoL scales for patients with PD could be divided into universal (general - generic) and specific for the disease (disease - oriented).

## Generic questionnaires

Among the generic questionnaires used in the other disease entities, the assessment of QoL in patients with PD mostly used are: Medical Outcome Study 36-Item Short Form Health Survey - SF - 36, EuroQol EQ-5D, Sickness Impact Profile (SIP) [**11-14**], Life Satisfaction Questionnaire - LSQ) [**15**], WHOQOL BREF and Quality of Well-Being Scale - QWBS [**13-19**].

The above-mentioned questionnaires have been tested in many countries. In literature, there are numerous and detailed data on their validity and reliability - also in relation to PD [**19**]. The scale of the SF-36 allows the assessment of the eighth areas of QoL during the four weeks preceding the survey; it takes about 9 min to be filled in. It is particularly useful in predicting the course of the disease [**20**]. The disadvantages include the effect of the lower and upper limit and a relatively low sensitivity to change QoL.

The EQ-5D scale allows the assessment of the fifth areas of health and self-esteem at the time of the study. Filling time is of 3 min. Because of the three levels evaluation, the EQ-5D system is poorly sensitive to QoL changes. It is primarily intended for managing healthcare - healthcare decision-makers [**14**].

Sickness Impact Profile (SIP) questionnaire allows the assessment of 12 areas of functioning at the time of study and (in contrast to SF-36 and EQ-5D) it is sensitive to the patient`s change. The disadvantages of SIP include its length (136 items) which means that the filling time can be up to 30 minutes [**15**]. LSQ is used to assess the overall satisfaction and satisfaction with the eighth areas of life. The answers to the sixth estimated point scale from very dissatisfied to very satisfied [**16**]. QWB allows the assessment of mobility, physical activity, social activity and 27 symptoms. The combination of the above categories can identify 43 functional levels of the patient. It is recommended that the questionnaire is completed by interview, by a person trained for that purpose. Filling time is between 10 - 15 min [**19**].

## Questionnaires specific for PD

Some questionnaires were developed exclusively for patients with Parkinson's disease (PD). The best known are two scales, Parkinson `s Disease Questionnaire (PDQ-39) and Parkinson` s Disease Quality of Life Questionnaire (PDQL)], are less frequently used scales than PDQUALIF PIMS. Martinez-Martin believes that the quality of life scales are more useful than other scales in evaluating the results of rehabilitation and drug treatment of Parkinson's disease or neurosurgery [**21**].

PDQ-39 was created in 1995 by a group of colleagues from Oxford University led by Peto and Jenkinson [**20**]. It evaluates 39 parameters in eight groups of issues. These are mobility, activities of daily living, emotional well-being, stigma of the disease (stigma), back in the next, cognitive, communication, bodily discomfort. Number of evaluated parameters in each group is from 3 to 10 The respondent has to choose one of five possible answers. Comparative studies PDQ-39 scales and SF-36 showed the highest sensitivity in the evaluation of PDQ mobility, activities of daily life, emotions and the stigma of the disease, and SF-36 in the assessment of physical condition and pain [**19,20**]. This means greater usefulness of the scale of which is a kind of PDQ-39 in the evaluation function in Parkinson's most dysfunctional. Authors are willing to provide scale, and, for a small fee, provide a detailed guide (manual). It appeared in several papers using the PDQ-39, including the Polish authors [**22**]. In 1997, Jenkins and colleagues published an abbreviated version of the PDQ to 8 PDQ-8 items [**23**].

PDQL scale described by de Boer et al. in 1996 consists of 37 items covering four areas [**15**]. These are Parkinsonian symptoms which also contain side effects of levodopa (14 items), symptoms called "systemic" (difficulty in walking, worse mood, disturbance of resting over the night, exhaustion, constipation, urinary incontinence) - 7 items; emotional sphere (9 items ) and social functions (hobbies, sex, recreation, leisure trips, Public Speaking (Difficulties signing your name public), transport difficulties, depressed mood and intimidation) -7 position. The test has the ability to choose one out of five answers on the incidence of a-disorders: 1) permanently, 2) most of the time, 3) sometimes (some of the time), 4) a little, 5) never. Statistical calculations have shown a correlation between symptoms of Parkinsonism and their effects on emotional and social [**15**]. This scale has long been restricted and it was only relatively recently that some reports of other authors were published [24-26]. 

In 1996, Calne et al. described the scale of the Impact of Parkinson's - Parkinson's Impact Scale (PIMS) [**27**]. The five-assessment system (0 = no change, 1 = low impact, 2 = moderate impact, 3 = average, a strong influence and 4 = strong influence) means that the patient assesses the impact of disease on the ten spheres of life. These are: 1) the phenomena of positive self-esteem (self-esteem, happiness, optimism), 2) self-assessment of negative effects (level of stress, anxiety or depression), 3) family relationships, 4) the relationship with the environment 5) Work: 6) departures from home (for work, meetings, etc.), 7) leisure and recreation, 8) security (avoiding injury), 9) financial security, 10) sex [**27**]. According to Marinus et al., PIMS scale compared with PDQ-39 scales has the smallest PDQL suitability for testing the quality of life in PD [**28**]. 

In 2003, Welsh et al. described the scale of the Quality of Life in Parkinson's Disease - Parkinson's Disease Quality of Life scale (PDQUALIF) [**29**]. It consists of 33 items covering eight domains: function and role in society, self-image (self-image), sexuality, sleep, look at the world (outlook), physical fitness, independence, urinary function, global assessment of quality of life (HRQOL). The evaluation system is a five-step (ladder Likert): from 0 to 5, the end result extends in a range from 0 to 128 points [**29**].

## Evaluation of cognitive functions and depression

Scores of QoL are usually supplemented by a study of cognitive function and depression, since these factors significantly affect the sense of quality of life, as well as an important context for the interpretation of test results QoL. Severe cognitive impairment and depressive symptoms may be a contraindication to test PRO. Scoring QoL in patients with PD, especially when it is done for scientific purposes, requires the measurement of the functional status and fatigue, because in addition to depression and cognitive impairment, they are the most important determinants of QoL in patients with PD. Following, a review of the most commonly used questionnaires to assess the emotional and cognitive functions is made. 

According to different authors, cognitive dysfunctions are observed in 40 - 65% of patients with PD. In preliminary diagnosis of these disorders, the most often used tests are: the Benton test, Mini Mental State Examination (MMS), Clock Drawing Test, Rosenbaum vision screening test (Pocket Vision Screener - PVS), Wechsler test [**3,25,30**].

The specific neuropsychological tests commonly used are: Controlled Oral Word Association Test (COWAT), California Verbal Learning Test (CVLT), Digit Symbol Modality Task (SDMT), Delis-Kaplan Executive Function System (D-KEFS), Paced Auditory Serial Addition (PASAT) [**24,26,28-29**]. 

We should also mention that, in addition to psychological research methods commonly used in other diseases, in Parkinson's disease, a rapid test used is the psychometric Mini-Mental Parkinson, described in 1992 by Mahieux et al. [**31**]. The psychological tests most commonly used in the PD and described by colleagues and Marinus are the following: scope-COG, SCOP-AUT14, SPES-motor-SCOPE, SCOPE-PS (Psychosocial) [**32**]. The negative impact of cognitive impairment on QoL was demonstrated in a number of studies [**33-36**]. 

Depression in PD is fairly well understood, its prevalence is estimated in 40 to 60% of the patients [**34-38**]. 

For the evaluation of depression in PD, the most frequently applied methods are Beck's Inventory, the Hamilton scale, Hospital Anxiety and Depression Scale (Hospital Anxiety and Depression Scale - HADS), Zung scale and the Montgomery-Asberg scale (Montgomery-Asberg Depression Rating Scale - MADRS [**28,34,36**]. Hamilton's scale is more useful for the study of depression in the elderly, while Beck's scale is often used in younger people; previously conducted studies clearly indicate the negative effect of depression on QoL patients. 

Depression is the most commonly explored mood disorder influencing quality of life in PD and has been found to be the best predictor overall for quality of life in several studies [**34-38**]. In a population-based survey using the Parkinson’s Disease Quality of Life Questionnaire (PDQ-39) and the Beck Depression Inventory (BDI), Schrag and colleagues [**36**] found that the factor most strongly related to poorer quality of life was depression, although motor disability was also significantly associated. In a model predicting PDQ-39 scores, the BDI score accounted for 54% of the variance, whereas motor disability scores accounted for only 15%. The Global Parkinson’s Disease Survey Steering Committee [**37**] also found the BDI score to be the most significant predictor of quality of life, accounting for 58% of the variance in PDQ-39 scores, whereas stage of motor severity and PD medication (levodopa, either alone or in combination with other dopaminergic drugs) together explained only 17% of the variability of quality of life in PD. Besides depression, anxiety disorders are a clinically significant problem in patients with PD. The prevalence of anxiety has typically been found to be of 20–46% of PD patients [**39-42**] though other studies have reported rates of up to 75% [**13**]. The number of patients with PD who experience significant anxiety is greater than that of individuals with other chronic medical conditions such as multiple sclerosis or of the general population [**42**]. Anxiety is thought to have an important impact on motivation, treatment compliance, and cognition and can exacerbate parkinsonian symptoms [**43**]. The contribution of anxiety to quality of life in PD has been less studied, although anxiety symptoms have been found to have a significant association with poorer quality of life in the general population [**44**].

## Global fatigue

Fatigue is one of the most common symptoms of PD and it is associated with reduced quality of life. It has been recently reported in literature that fatigue in PD has an increasing frequency. This can be defined as uncontrollable apathy, lack of energy or feeling exhausted with no link to depression, or muscle weakness [**45-48**]. 

 That is why, for the evaluation of fatigue, more than 30 scales have been developed.

 The most frequently used are the Fatigue Severity Scale – FSS by Krupp et al. and the Modified Fatigue Impact Scale (Modified Fatigue Impact Scale - MFIS). FSS is composed of nine items, to which the patient responded up to a 7-point scale estimation. The final result is the arithmetic mean of the scores of all items. The average score of FSS for patients with MS is of 6.5 [**49**]. MFIS is a modification of the scale Fatigue Impact Scale by Fisk et al. It contains 21 items, concerning the impact of fatigue on mental, physical, and social functioning. The final result is the sum of points from the scale of individual items [**45**].

 The problem of fatigue in patients with PD is frequently underestimated, even though as many as every third patient considers it the most disabling symptom, which is manifested in the Health-Related Quality of Life (HRQL) measured by using Parkinson's Disease Questionnaire (PDQ-39) and Short-Form 36 (SF-36) [**46**]. Hitten et al. observed excessive fatigue in 48% of patients with PD [**47**]. Fatigue is frequently (though not always) associated with depression and is dependent on it, which is proved in the following psychological tests: Multidimensional Fatigue Inventory (MFI) and Geriatric Depression Scale (GDS). In the Norwegian population-based study, fatigue was found in 44% of all 232 patients included [**48**]. 

 Fatigue is independent of motor and non-motor symptoms of PD such as pain, night sleep disorders, and daytime sleepiness. A dose of L-DOPA, duration of dopaminergic treatment, presence, or absence of motor fluctuations, as well as the severities of PD, evaluated with the Unified Parkinson’s Disease Rating Scale, were found to have no relation with fatigue [**49-50**].

 Fatigue seems to be partly subject to L-DOPA treatment. Objectively measured muscle strength showed an increasing tendency after administration of L-DOPA with carbidopa, independently of the Multidimensional Fatigue Inventory scale (MFIS) result [**51**]. 

## Sleep disorders

Studies showed that 60-98% of the patients with PD suffer from sleep disorders [**52-55**]. Despite such high incidence, sleep disorders are not included in the routine clinical examinations. The underlying illness and neurotransmission disorders are the most common factors disturbing sleep pattern in patients with PD. Primary sleep disorders associated with disturbed dopaminergic transmission in the CNS, occurring in patients with PD more commonly than in the overall population, are among other the following: periodic limb movement in sleep (PLMS) and REM sleep behaviour disorder as well as frequently occurring sleep apnoea syndrome [**55**]. PLMS and RLS occur in about 15-20% of PD patients [**56**].

 Sleep disorders, such as i.e. excessive daytime sleepiness, result in serious consequences like car accidents or impairment of social functioning, and thus a significant decrease of the PD patients’ quality of life. Unfortunately, little is still known about the influence of sleep disorders on life quality, since only a few authors have so far tried to conduct such studies. One of them was the prospective study by Happe et al., being a part of the FAQT project (Determinants of Quality of Life of Parkinson’s Disease Patients in the Ambulatory Care), which included 209 patients [**57-58**]. The quality of life was lower in a group of patients with sleep disorders and differed depending on their intensity. Patients who developed more significant sleep disorders during the year of the study showed a lower number of points in all three areas of life quality (mental health, physical health and general health). Patients who suffered from sleep disorders less frequently than at the beginning of the study, achieved better results in the evaluation of life quality [**57-58**].

 Polysomnography is an objective method used for diagnosing sleep disorders that allow the evaluation of sleep apnoea. There are currently no easily available diagnostic screening tests for sleep disorders in PD. The Epworth Sleepiness Scale (ESS) or Pittsburgh Sleep Quality Index (PSQI) does not evaluate different types of sleep disorders in patients with PD. The Unified Parkinson’s Disease Rating Scale (UPDRS) contains only one question concerning sleep disorders, while Parkinson’s Disease Quality of Life Scale (PDQ39) only two of them. However, most cases of such disorders in which the Epworth Sleepiness Scale is used, evaluate excessive daytime sleepiness. The questionnaire consists of 8 points referring to 8 different everyday situations and the probability of falling asleep. The subject may score 0-3 points for each question (3 corresponds to a high chance of dozing) [**59**]. The result >10 indicates that the subject suffers from daytime sleepiness, while >15 suggests that the sleepiness is excessive [**59**]. A result higher than 16 points indicates a severe sleepiness and high possibility of sudden, unexpected episode of falling asleep during daytime [**59**]. Recently, a new scale was introduced for the evaluation of different types of sleep disorders in PD – Parkinson’s Disease Sleep Scale (PDSS). It is a simple scale used for the conduction of screening tests for different types of sleep disorders in PD, indicating a possible need for the conduction of specialist examinations of sleep patterns and allowing the introduction of a new treatment. It consists of 15 questions concerning the most common symptoms of sleep disorders. The form is filled in by the patient who is asked to mark a point at a 10cm line corresponding to the frequency or intensity of particular symptoms. The researcher interprets the result by crosschecking the lines with a transparent scale. Each question may be scored from 0 (frequent and intensive symptoms) to 10 (no symptoms). The maximum test score is 150 (lack of any symptoms indicating sleep disorders). Authors recommend that the PDSS and ESS are routinely used in clinical studies of patients with PD [**60**]. Another scale – the Sleep Disorders Questionnaire – consists of 175 questions concerning 4 different types of sleep disorders (sleep apnoea, narcolepsy, psychiatric sleep disorders and periodic leg movements) but is not frequently used [**61**].

Appendix

 Parkinson`s Disease Questionnaire (PDQ-39)

 Mobility: 

 Difficulty doing leisure activities

 Difficulty looking after your home

 Difficulty carrying bags of shopping

 Problems walking half a mile

 Problems walking 100 yards

 Difficulty getting around the house

 Difficulty getting around in public places

 Needed to be accompanied when out

 Frightened or worried about falling in public

 Been confined to the house more than liked

 Activities of daily living: 

 Difficulty washing yourself

 Difficulty dressing yourself

 Problems doing up buttons or laces

 Problems writing clearly

 Difficulty cutting up food

 Difficulty holding a drink

 Emotional well being:

 Felt depressed

 Felt isolated and lonely

 Felt weepy or tearful

 Felt angry or bitter

 Felt anxious

 Felt worried about the future

Stigma:

 Felt you had to conceal PD

 Avoided eating or drinking in public

 Felt embarrassed by having PD

 Felt worried by others` reaction to you

 Social support:

 Problems with close relationships

 Not had support from spouse or partner

 Not had support from friends or family

 Cognitions:

 Unexpectedly fallen asleep during day

 Problems with concentration

 Felt your memory was bad

 Distressing dreams or hallucinations

 Communication:

 Difficulty with speech

 Felt unable to communicate properly

 Felt ignored by people

 Bodily discomfort:

 Painful muscle cramps or spasms 

 Aches and pains

 Felt unpleasantly hot or cold

 Responses:

 never (0)

 occasionally (1)

 sometimes (2)

 often (3)

 always (4)
